# Addressing Potential Researcher Distress in Nurse‐Led Research: Ethical Considerations and Practical Strategies

**DOI:** 10.1111/jan.16799

**Published:** 2025-02-03

**Authors:** Carmel Bond, Adrianna Watson, Debra Jackson

**Affiliations:** ^1^ Sheffield Hallam University Sheffield UK; ^2^ Brigham Young University Provo USA; ^3^ University of Sydney Sydney Australia

**Keywords:** moral distress, nurse researcher, nursing research, secondary traumatic stress, vicarious traumatisation

## Abstract

**Aims:**

To discuss the need for nurse researchers to consider to the potential for psychological distress when conducting studies on sensitive topics.

**Design:**

Discursive paper.

**Methods:**

Drawing from existing literature, we highlight the ethical obligations of researchers to recognise and manage their emotional responses, especially as these can potentially lead to burnout and re‐traumatization. In this paper, we propose practical strategies to mitigate these risks, including trauma‐informed practices, peer support systems, structured mentorship and the establishment of vicarious trauma (VT) plans.

**Conclusion:**

Prioritising researcher well‐being in nursing research is essential for ethical practices and the mental health of those involved in undertaking research in sensitive areas.

**Implications for the Profession and/or Patient Care:**

Support strategies, such as formal team debriefings, resilience training, VT plans and peer support, can foster safer and healthier research environments, when researching in sensitive areas.

## 1 Introduction

In academic and clinical research, ethical considerations rightfully and traditionally focus on protecting research participants' rights, safety and well‐being (Kandi and Vadakedath 2022). However, there is less ethical oversight relating to researcher well‐being (Nguyen et al. 2021; Silverio et al. 2022), specifically regarding the potential for psychological distress among researchers. Nurse researchers may encounter emotional challenges when engaging with sensitive or distressing topics, such as painful and distressing lived experiences related to health (Nguyen et al. 2021).

In this paper, we argue that nurse researchers have an ethical obligation to consider the potential for psychological distress, vicarious trauma (VT) and secondary traumatic stress, especially when conducting qualitative studies on sensitive topics (Figley 1995; Nguyen et al. 2021). The terms VT and secondary traumatic stress (STS—also known as secondary trauma) refer to the negative consequences an individual experiences as a result of having been exposed to other people's trauma (Pellegrini, Moore, and Murphy 2022). This ethical obligation must therefore extend to all of those involved in engaging with the data, including people undertaking transcription (Wilkes, Cummings, and Haigh 2015) and those working with secondary data (Jackson 2013). As such, we explore the issue of researcher distress, position this as a key ethical issue in nursing research and offer practical strategies for recognising and mitigating the emotional burden that may accompany such work. Identifying and planning to mitigate the possibility of VT and/or secondary traumatic stress in research is essential to ensure the well‐being of researchers. Therefore, maintaining the mental health and well‐being of researchers, as well as building personal resilience will enable quality nurse‐led research outcomes to be maintained in the future.

## 2 Neglecting Researcher Well‐Being: An Ethical Oversight

Research ethics committees (RECs) are established to safeguard the interests of research project participants, ensuring they are protected from harm, coercion and exploitation (Kaplan et al. 2023). These committees are also responsible for overseeing the integrity of the research process itself. However, it is equally important to safeguard the mental health of members of research teams within that process. While researcher well‐being is at least partially addressed in some parts of the world, many RECs seldom consider well‐being support for those conducting the research, particularly when the research topic involves sensitive, emotionally charged or potentially traumatising subject matter (Drozdzewski and Dominey‐Howes 2015). This lack of focus on researcher well‐being within the ethical review process creates a vulnerability that must be addressed.

Addressing this gap is crucial for fostering resilience and supporting the mental health of researchers, who may otherwise face a cumulative effect of burnout and compassion fatigue due to sustained exposure to trauma in their research, as well as in their professional role (Cooper et al. 2020). As Kaplan et al. (2023) note that a general focus on safeguarding participants over providing protection for researchers is an ongoing global issue, which transcend disciplines, and is underpinned by a lack of training for research staff before they enter the field. This is particularly pertinent for post‐graduate researchers (PGRs), and early career researchers, as research findings have highlighted a growing concern regarding the mental health of PGRs, particularly doctoral researchers (Evans et al. 2018). However, the sources of stress within this group have largely overlooked how the research process itself may contribute to the high prevalence of mental distress (Byrom et al. 2020; Nguyen et al. 2021).

As researchers, nurses often study complex, distressing, and highly sensitive themes such as chronic illness, end‐of‐life care, mental health, family violence and patient trauma (Buur et al. 2024; Dangerfield, Anderson, and Tinnell 2024). These topics frequently require the exploration of personal and often painful experiences of participants, exposing researchers to emotional and psychological distress (Jackson, 2013; Nguyen et al. 2021). Likewise, nurse researchers are often motivated by their chosen subject, which they may have a personal interest in and/or experience of. This can potentially make them susceptible to distress, as they themselves may have had experiences similar to their research participants. This can lead to researchers becoming re‐traumatised. Re‐traumatization occurs when something in a present experience is reminiscent of past trauma, leading to the person being traumatised again or even creating a new trauma (Sweeney et al. 2018).

Re‐traumatization can be an issue in studies involving sensitive topics, where the emotional and psychological well‐being of participants must be carefully considered to avoid triggering a response that brings a previous traumatic memory into the here‐and‐now; for example, feelings, thoughts, sensations or images (Conolly et al. 2023). Hence, researchers must plan for and implement strategies to mitigate these risks, ensuring a supportive environment for participants as well as for themselves throughout the research process.

## 3 Personal Distress in Nurse‐Led Research

**FIGURE 1:**
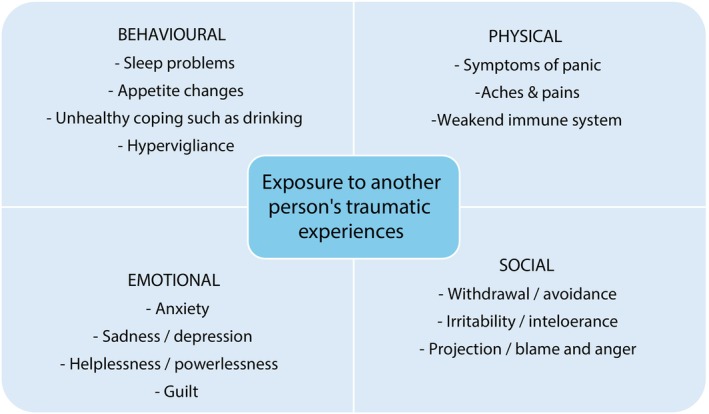
Some of the potential individual responses as a result of exposure to another person's trauma.

Nurse researchers are uniquely positioned at the intersection of clinical practice and academic inquiry, which often involves engaging with participants' deeply personal (sometimes traumatic) lived experiences. While understanding these experiences is invaluable for advancing knowledge in nursing and healthcare, they can also lead to personal distress (Morrison and Joy 2016). This exposure can lead to VT, which refers to cognitive and emotional impacts of working with people who have experienced trauma (Isobel 2021). VT is widely reflected in the literature and typically relates to clinical settings and where professionals are exposed to distressing experiences, such as child abuse, sexual assault, interpersonal violence and traumatic deaths (Leung, Schmidt, and Mushquash 2023). However, as San Roman Pineda et al. (2023) indicate, there remains a dearth of research relating to the trauma‐related experiences of researchers. Researchers who do write about their experiences of VT, because of researching distressing topics, are qualitative researchers (Eades et al. 2021). Although, San Roman Pineda et al. (2023) note that quantitative researchers may still be prone to research‐related trauma and all researchers should be trained in how to handle it. Anyone who is exposed to the traumatic events of others can experience psychological distress, and the personal response may be different for each individual person. However, typical responses are often characterised by a range of feelings, including fearfulness, helplessness, irritability, not wanting to engage with work and avoidance of traumatic stories, as well as intrusive thoughts and flashbacks (Runyon, Copel, and Trout 2024). Depending on the research area, nurse researchers are at a significant risk of experiencing distress when carrying out their research. It is therefore important to be aware of the various signs of distress—Figure 1.

Studies on vicarious traumatization (or STS) among nurses have shown high prevalence rates, particularly in emergency and paediatric care, where over 60% of nurses report experiencing chronic symptoms (Alshammari et al. 2024; Ariapooran, Ahadi, and Khezeli 2022; Duffy, Avalos, and Dowling 2015; Xu et al. 2024). While this issue is widely recognised in clinical practice, nurse researchers encounter similar challenges when they engage with participants' traumatic experiences (Silverio et al. 2022). Given the potential for these adverse effects, it is critical to develop frameworks for addressing and mitigating researcher distress as part of researcher training and the research process.

Although any researcher can experience VT, many health and social care workers are regularly exposed to human suffering and distress. However, the emotional impact of this is often underestimated (Leung, Schmidt, and Mushquash 2023), partly due to the assumption that registered nurses and other healthcare professionals, who are accustomed to dealing with distressing situations in clinical practice, are inherently resilient (Cooper et al. 2020). It is important to understand that while clinical training may prepare nurses for the emotional challenges of patient care, ‘being with’ participants as they share stories of pain (and later analysing the data and being immersed in the storied accounts) can provoke a different type of emotional strain, one that warrants specific ethical and practical considerations. Therefore, as considered by Taylor et al. (2016) recognising the potential for researcher trauma is the first step toward addressing the ethical gap in researcher well‐being. Practical strategies can be implemented to mitigate the risk of emotional harm and ensure that researchers are supported throughout the research process.

## 4 Proactive Measures to Support Nurse Researchers

Nurse researchers often work with emotionally charged and highly sensitive topics that can lead to burnout and emotional exhaustion if not managed effectively (Nguyen et al. 2021; Taylor et al. 2016; Cooper et al. 2020). To address the challenges of vicarious traumatization among nurse researchers, it is essential to implement practical strategies that prioritise their mental health and resilience. Institutions and research organisations can adopt a multifaceted approach to provide comprehensive support for nurse researchers, which may include methodological measures to mitigate VT, mental health resources, personal resilience training, structured support systems such as formal team debriefing strategies and VT plans (Taylor et al. 2016).

Despite the size of the team, it is important to consider ways to support researchers so that the potential risks of distress can be mitigated. Regular supervision and peer support systems are essential, allowing researchers to debrief, share emotional challenges and receive guidance from more experienced colleagues. Researchers may consider prioritising trauma‐informed practices, ensuring that they and their teams are equipped to recognise signs of emotional distress both in themselves and participants (Isobel 2021). Although it is unclear whether VT and/or secondary stress can be remedied by a trauma informed approach in this context of undertaking research, trauma informed approaches have been shown to increase positive outcomes for patients and for those who provide care (Barnhill et al. 2019; Hales et al. 2019). Goodwin and Tiderington (2022) discuss the importance of trauma‐informed approaches in social work, emphasising self‐care and the establishment of safe, respectful environments, for both researchers and participants, to prevent re‐traumatization. Therefore, encouraging researchers to identify and name any distress, at the very least, may go some way to reducing potential negative consequences of hearing about someone else's' traumatic experiences.

Researchers can also incorporate emotional reflexivity into their methodology (Jackson, Backett‐Milburn, and Newall 2013), routinely reflecting on their emotional responses and adjusting their approach as needed to recognise the role of the self in the research process. In their secondary analysis of child health data in which accounts of physical and sexual abuse were disclosed, Jackson, Backett‐Milburn, and Newall (2013), acknowledged that the research process was disrupted by the occurrence of distressing emotional states, such as sadness, anger and horror, and the requirement of the team to manage these emotions. This team drew on the concept of emotional reflexivity to support the wider research team and maintain the quality of data analysis (Jackson 2013). Conolly et al. (2023) elected to prioritise and integrate participant autonomy and researcher reflexivity into their research framework to lessen the likelihood of distress and trauma for both research participants and researchers, who were collecting potentially distressing data.

Also, employing strategies such as emotional self‐regulation techniques and setting clear boundaries with participants can help maintain professional distance, ensuring researchers can remain focused on ethical considerations while safeguarding their own well‐being (Kraiss et al. 2020). By integrating these proactive measures, qualitative researchers can mitigate the emotional toll of their work and sustain both the quality of their research and their own personal resilience.

### 4.1 Resilience Training

Cooper et al. (2020) outline the benefits of resilience training programs, which are particularly valuable for nurse researchers as they provide tools and strategies to enhance emotional wellbeing. Such training may focus on stress management, mindfulness practices, and techniques for building psychological resilience, which are beneficial for those regularly exposed to distressing narratives. By building resilience, nurse researchers may better withstand the emotional demands of their work, reducing the likelihood of experiencing compassion fatigue and burnout. Buur et al. (2024) argue that resilience training should be a continuous initiative, seamlessly embedded into the research process and tailored to meet the unique needs of those engaging with trauma‐related topics, for example, mindfulness practices like meditation and breathing exercises can help researchers maintain emotional balance, while cognitive‐behavioural strategies may aid in reframing stressful experiences.

### 4.2 Structured Support and Mental Health Check‐Ins

Establishing structured support systems within research institutions is another crucial strategy. Silverio et al. (2022) emphasise the importance of systems like peer support groups, mentorship programs, and dedicated research supervision aimed at fostering emotional well‐being. Peer support groups offer nurse researchers a supportive environment to discuss their experiences and manage the unique emotional challenges inherent to their work. Such groups facilitate mutual support, reduce feelings of isolation and allow researchers to discuss coping strategies in a collaborative environment. Mentorship programs that pair experienced nurse researchers with less experienced ones can also offer guidance and support, particularly for managing stress related to VT (Cooper et al. 2020). Mentorship enables knowledge sharing and allows mentors to model strategies for building resilience, helping newer researchers develop coping skills.

Embedding strategies to reduce or prevent and identify potential for traumatization early can be very useful. In a study that involved non‐participant observation of verbal abuse directed at nurses in the work environment, the possibility of VT was identified and so the research team established regular telephone contact with colleagues who were out if the field and made debriefing or counselling available (Jackson et al. 2013). These authors reported two occasions during data collection where debriefing was necessary but stated that no further counselling was required (Jackson et al. 2013).

For research students and early career researchers, supervision that emphasises emotional health is another valuable element of support. Supervisors can help new researchers recognise signs of VT early on, guiding them toward appropriate resources and interventions. Regular check‐ins with supervisors can provide ongoing emotional support, ensure that researchers remain aware of their mental health status and help mitigate the potential for emotional overload (Buur et al. 2024). Integrating mental health check‐ins as a standard part of research supervision can help normalise discussions around mental well‐being, reducing stigma and promoting a culture of self‐care.

## 5 Mental Health Resources

Providing accessible mental health support and resources is also an effective way to support nurse researchers. For example, resources such as counselling services, mental health workshops, and access to trained mental health professionals can help researchers process and manage the emotional burden of engaging with traumatic content. Mental health resources should be tailored for researchers, emphasising that institutions should promote these services and normalise their use within the research community (Silverio et al. 2022). Additionally, confidential counselling services can offer a safe space for researchers to discuss their experiences and develop coping mechanisms for managing the effects of VT and secondary traumatic stress (Byrom et al. 2020).

Rennick‐Egglestone et al. (2019) outlined several approaches to addressing researcher distress due to VT when engaging deeply with participant stories of mental health recovery. The team implemented regular debriefing sessions and consultations with a Lived Experience Advisory Panel (LEAP). The LEAP consisted of individuals with prior mental illness and/or mental health difficulties; the group offered feedback regarding the emotional and psychological aspects of the research. This collaborative approach was intended to reduce isolation and provide peer support, essential for mitigating the emotional impact of recovery narratives on researchers, particularly as the researchers were engaging in repetitive reading of sensitive, trauma‐related materials. However, this approach may inadvertently place burden upon a group who may themselves be re‐traumatised through supporting potentially traumatised researchers. For clinical academic nurses, there may be an opportunity to engage in a structured peer debriefing session, away from the employing organisation, which has been shown to be useful in supporting nurses' who were experiencing distress during the COVID‐19 pandemic (Bond et al. 2022).

In addition to structured mental health support, promoting resilience among nurse researchers through peer support and trauma‐informed policies can be equally beneficial. For instance, in a study on homicide, researchers highlighted the value of trauma‐informed support systems, including regular check‐ins, flexible work schedules, and peer debriefing sessions, which allowed researchers to manage VT while sustaining engagement with challenging content (AbiNader et al, 2023). Establishing formal and informal peer‐support structures can help mitigate isolation and create a supportive environment where researchers can discuss difficult experiences with colleagues who understand their work's unique demands. These peer interventions are essential for fostering resilience, as they offer both practical and emotional support, enabling researchers to process their experiences without feeling detached or overwhelmed. Integrating these approaches in nursing research teams can provide a well‐rounded support system, promoting both mental health and professional sustainability.

## 6 Vicarious Trauma Plans

Researchers and RECs should consider the benefits of a ‘vicarious trauma plan’ to assist in the early recognition and mitigation of psychological distress and trauma. Taylor et al. (2016) presented a framework for VT in research to help researchers recognise projects in which it was more likely to occur, and also highlighted the importance of having a plan to address VT in research.

A VT plan is a structured approach aimed at helping researchers recognise, manage and prevent the negative effects of the VT that can occur when exposure to research participants' traumatic experiences leads to emotional distress or psychological harm. Key components of a VT plan can include raising awareness about the signs of VT and its impact on mental health, strategies to mitigate trauma, such as regular supervision and mentorship, and debriefing sessions that provide a safe space for researchers to reflect on their own emotional responses and reactions. This approach can also encourage self‐care strategies, such as maintaining a healthy work‐life balance, establishing emotional boundaries between researchers and participants and understanding the role of self in the research process.

We propose that a VT plan including access to professional counselling or other support external to the research team (such as student counselling services) should always be in place for students and PGRs as they are often new to research and an effective plan can help to mitigate potential for emotional burden, reduce the risk of burnout and enhance the quality of research. This is particularly important for students working methodological spaces or certain subject areas where they are likely to be exposed to distressing materials.

Examples of research projects that would benefit from a VT plan include studies involving in‐depth interviews with survivors of trauma, such as domestic violence, sexual assault, or natural disasters, where researchers are repeatedly exposed to detailed accounts of distressing experiences. Other projects that involve analysing sensitive case files—such as those on child abuse, criminal violence or serious medical conditions—can lead to cumulative stress and VT, making a formal support plan essential.

Conversely, a VT plan may not be necessary for studies involving minimal emotional engagement with participants, such as research on more neutral topics (e.g. dietary preferences or exercise habits), where there is limited risk of psychological distress for researchers. Similarly, studies where researchers analyse de‐identified data on non‐sensitive topics, without direct engagement with distressing content, may not require the same level of trauma support. However, research teams should still promote general mental health resources to ensure well‐being across all project types.

## 7 Conclusions

In conclusion, addressing researcher well‐being in nursing research is essential to ensure both ethical research practices and the mental health of those conducting research on sensitive topics. Although RECs, rightly, prioritise participant welfare, the emotional impact on researchers remains insufficiently considered, which is pertinent for nurse researchers, who regularly encounter traumatic or distressing narratives, particularly when undertaking qualitative research. Implementing a supportive framework that includes strategies like peer support, resilience training, structured supervision, and VT plans can provide essential support for nurse researchers. Such measures can mitigate the risks of burnout and VT, allowing researchers to engage ethically and sustainably with sensitive topics while maintaining their personal well‐being and the quality of their work. Addressing these concerns will foster a healthier research environment and support the longevity of nurse‐led research contributions.

## Conflicts of Interest

The authors declare no conflicts of interest.

### Peer Review

The peer review history for this article is available at https://www.webofscience.com/api/gateway/wos/peer‐review/10.1111/jan.16799.

## Data Availability

No new data has been created from this paper.
